# Loneliness, Methamphetamine Use, and Cardiovascular Risk Factors Among Sexual Minority Men in the COVID-19 Era

**DOI:** 10.1007/s12529-024-10288-0

**Published:** 2024-04-29

**Authors:** Emily J. Ross, Daniel E. Jimenez, Delaram Ghanooni, Armando Mendez, Sabina Hirshfield, Keith J. Horvath, Britt DeVries, Samantha E. Dilworth, Adam W. Carrico, Claudia A. Martinez

**Affiliations:** 1 Department of Public Health Sciences, University of Miami, 1120 NW 14th Street, Miami, FL 33136, USA; 2 Department of Psychiatry and Behavioral Sciences, University of Miami Miller School of Medicine, 1120 NW 14th Street, Suite 1436, Miami, FL 33136, USA; 3 Department of Public Health Sciences, Miller School of Medicine, University of Miami, 1120 NW 14th Street, Miami, FL 33136, USA; 4 Division of Endocrinology, Diabetes and Metabolism and the Diabetes Research Institute, Department of Medicine, University of Miami Miller School of Medicine, 1450 NW 10 Ave, Miami, FL 33136, USA; 5 Department of Medicine, STAR Program, SUNY Downstate Health Sciences University, 450 Clarkson Avenue, Brooklyn, NY 11203, USA; 6 Department of Psychology, San Diego State University, Alvarado Court, San Diego 6363, CA, USA; 7 Department of Medicine, Center for AIDS Prevention Studies, University of California San Francisco, 550 16 Street, San Francisco, CA 94158, USA; 8 Cardiovascular Division, Department of Medicine, University of Miami Miller School of Medicine, 1120 NW 14th Street, Miami, FL 33136, USA; 9 Department of Cardiology and Interventional Cardiology, University of Miami Miller School of Medicine, 1120 NW 14th Street, Miami, FL 33136, USA

**Keywords:** Loneliness, Social isolation, HIV, Stimulant use, Sexual minority health

## Abstract

**Background:**

Important gaps exist in our understanding of loneliness and biobehavioral outcomes among sexual minority men (SMM), such as faster HIV disease progression. At the same time, SMM who use methamphetamine are approximately one-third more likely than non-users to develop cardiovascular disease. This study examined associations of loneliness, stimulant use, and cardiovascular risk in SMM with and without HIV.

**Method:**

Participants were enrolled from August 2020 to February 2022 in a 6-month prospective cohort study. The study leveraged self-report baseline data from 103 SMM, with a subset of 56 SMM that provided a blood sample to measure markers of cardiovascular risk.

**Results:**

Loneliness showed negative bivariate associations with total cholesterol and LDL cholesterol in the cardiometabolic subsample (*n* = 56). SMM with methamphetamine use (*t*(101) = 2.03, *p* < .05; *d* = .42) and those that screened positive for a stimulant use disorder (*t*(101) = 2.07, *p* < .05; *d* = .46) had significantly higher mean loneliness scores. In linear regression analyses, negative associations of loneliness with LDL and total cholesterol were observed only among SMM who used methamphetamine.

**Conclusion:**

We observed lower cholesterol in SMM reporting loneliness and methamphetamine use. Thus, in addition to the observed associations of loneliness with cholesterol, there are important medical consequences of methamphetamine use including cardiovascular risk, higher HIV acquisition risk and progression, as well as stimulant overdose death. This cross-sectional study underscores the need for clinical research to develop and test interventions targeting loneliness among SMM with stimulant use disorders.

## Introduction

It is widely recognized that loneliness—a pervasive public health concern characterized by a subjective perception of social isolation or distress stemming from the discrepancy between one’s desired and actual social relationships—has notable effects on cerebral and cardiovascular health [1, 2]. It is proposed that loneliness has a downstream effect through poor health behaviors, heightened stress response, and inadequate physiological repairing activity, i.e., poor reparative processes amidst chronic stress [3]. Through these mechanisms, loneliness alters inflammatory and metabolic markers [4], as well as chemokines, immunoglobins, and stress circuitry [3]. The clinical relevance of these alterations is supported by research documenting that loneliness predicts mortality, morbidity, accelerated physiological aging, cardiovascular risk, depression, and cognitive decline [5–9].

Greater vulnerability to loneliness is an important, modifiable determinant of health disparities [10]. Sexual minority individuals, including lesbian, gay, bi + (bisexual, pansexual, fluid), and asexual persons [11], experience higher rates of loneliness compared to heterosexual peers with estimates ranging between 13 and 35% [12–16]. In addition, sexual minority individuals that report greater loneliness are at a higher risk of experiencing perceived discrimination, stigma, and inequitable health outcomes, including unmet medical needs and worse health status [17, 18].

Sexual minority men (SMM) experience higher levels of stress associated with stigmatization as well as adverse views of oneself [19]. Higher stress levels among SMM are attributed to chronic exposure to a hostile society with highly negative attitudes toward sexual minority individuals—i.e., internalized heterosexism [20]. These are suggested factors related to concealment and isolation [21, 22]. Higher level of stress and isolation among SMM may also lead to marginalization and negative coping mechanisms such as substance use, including stimulants such as methamphetamine and cocaine/crack [23]. Notably, stimulant use is associated with higher levels of perceived loneliness as well as negative psychological and physiological health outcomes including greater cardiovascular risk among SMM [22]. Loneliness in SMM may be further exacerbated by experiencing adverse parental and familial reactions such as rejection, primarily originating from negative parent–adolescent relationships toward sexual minority youth [24]. The convergence of a hostile societal environment with chronic discriminatory experiences and limited familial support often culminates in a migration pattern among sexual minorities [25, 26]. This migration typically directs individuals toward urban settings, where they may encounter lower socioeconomic conditions, heightened prevalence of violence, and increased engagement in substance use, including stimulant use. Additionally, urban environments may foster higher levels of sexual risk behaviors and an elevated risk of HIV seroconversion. These interconnected elements are known to contribute to elevated rates of social isolation and loneliness, as evidenced in previous studies [25, 26].

Regardless, the COVID-19 pandemic brought greater social isolation across the globe due to social distancing measures. A recent cohort study investigated the impact of social distancing on feelings of loneliness among sexual minority individuals compared to cis-heterosexual individuals [27]. The results indicated that sexual minority participants experienced higher levels of loneliness than their cis-heterosexual counterparts. This trend was also seen for depressive symptoms [27]. Another COVID-19 study found a positive correlation between loneliness and C-reactive protein (CRP) levels—a marker of systemic inflammation—even after controlling for prior psychological distress and other relevant covariates [28]. However, this study did not account for differences across sexual orientation or gender identity status. Despite the greater prevalence of loneliness reported among sexual minority individuals, important gaps remain in our understanding of the association between loneliness and biobehavioral outcomes [14].

Among SMM, loneliness has also been associated with increased likelihood of engaging in substance use (including stimulants such as methamphetamine) [29–31]. In turn, methamphetamine and other stimulants also substantially amplifies risk for HIV, transmission, acquisition, and HIV disease progression [32, 33]. Furthermore, SMM who use stimulants are approximately one-third more likely than non-users to develop cardiovascular disease [34]. Additionally, data shows that methamphetamine users experience greater loneliness compared to non-users, even when controlling for HIV status and other covariates such as health-related behaviors [30]. The current study adapted Pourriyahi et al.’s framework of loneliness for the conceptualization of these relationships [3]. Specifically, Pourriyahi et al. view loneliness as an immunometabolic syndrome, emphasizing that loneliness is not just a psychosocial phenomenon, but instead involves immunologic, metabolic, and biobehavioral manifestations and pathways [3]. Guided by this framework, the current study explored loneliness among SMM within the context of stimulant use and cardiovascular risk.

The overarching goal of the present study was to examine the associations of loneliness, stimulant use (methamphetamine, crack/cocaine, or any stimulant use), and cardiovascular risk in SMM with and without HIV using a cross-sectional design. We examined whether loneliness was directly associated with screening positive for stimulant use disorder and markers of cardiovascular risk. We also hypothesized that the associations of loneliness with markers of cardiovascular risk would be less pronounced among SMM who use methamphetamine given its well-documented associations with decreased appetite and weight.

## Method

Participant baseline data from 103 SMM was available from a 6-month prospective cohort study in Miami-Dade and Broward Counties, Florida. Participants were enrolled from August 2020 until February 2022. Using a cluster sampling method, this study purposefully enrolled relatively equal numbers of SMM with and without HIV stratified by stimulant use. SMM were eligible for the study if they were 18 years old, identified as a cisgender male, were fluent in the English language, and reported engaging in anal sex with a cisgender male over the previous calendar year. Participants were recruited through social media platforms, including Grindr. Subsequently, we tracked each participant for a period of 6 months. Survey questionnaires were administered at baseline, 3-month, and 6-month intervals via Zoom. In addition to these remote assessments, we conducted two in-person visits during the study to collect urine and plasma biospecimen to measure stimulant use and markers of cardiovascular risk. This project was approved by the University of Miami Institutional Review Board with a certificate of confidentiality from the National Institute on Drug Abuse. All participants provided in-person informed consent and completed an interview via Zoom. Due to the study being conducted during the COVID-19 pandemic, both participants and the research coordinator were physically located on the same floor but in separate rooms when conducting the in-person informed consent process, which took place via the Zoom application with the research coordinator directly witnessing the procedure. Participants subsequently completed an online survey including all sociodemographic and self-report measures. The study leveraged self-report baseline data from 103 SMM with and without HIV recruited in South Florida. Data was available for a subset of 56 SMM who attended the baseline in-person biospecimen visit to provide a peripheral venous blood sample to measure markers of cardiovascular risk.

### Measures

#### Sociodemographic Factors and HIV Status

Demographic variables included age, race and ethnicity, level of education and income, sexual orientation status, HIV status, and time living with HIV (if HIV positive). HIV-positive status was measured via blood samples to measure HIV-1 viral load. HIV-negative status was measured via Oraquick ADVANCE^®^ Rapid HIV-1/2 antibody test. Participants were also asked to report time living with HIV as well as their antiretroviral therapy (ART) regimen.

#### Loneliness

Loneliness was measured using the UCLA loneliness measure [35]. The current study used a modified version consisting of 10 questions on a 4-point Likert scale which asks a series of questions such as “How often do you feel close to people?” and “How often do you feel isolated from others?” [35]. The UCLA’s loneliness measure Cronbach’s alpha for this sample was *α* = .89. Higher scores indicate greater experienced loneliness. The measure has five items which are negatively worded, and their scores were reverse-coded. Loneliness is measured by calculating the summary score across the items. There are no standardized clinical cut-offs, although previous research has used a score of < 20 as less than average loneliness, 20 to 24 as average to higher loneliness, 25 and above as high loneliness, and a score above 30 as very high loneliness [35–37]. Loneliness related to COVID-19 was assessed using a single-item: “How lonely do you feel now compared to how lonely you felt before COVID-19?” This question was presented on a 5-point Likert scale from 1: “I feel much more lonely since COVID-19” to 5: “I feel much less lonely.” This item was also reverse-coded.

#### Substance Use

Recent stimulant use was defined as any stimulant, methamphetamine, and cocaine/crack use reported on a modified version of the ASSIST or urine toxicology results reactive for cocaine or methamphetamine metabolites over the previous 3 months [38]. Stimulant risk severity for cocaine and methamphetamine use was calculated for participants that scored in the moderate-to-severe range, screening positive for a stimulant use disorder (i.e., low-risk versus moderate-to-severe risk). Recent tobacco use (over the previous three months) was also measured using the ASSIST.

#### Cardiovascular Risk Factor Variables

In a subsample of 56 participants with baseline data, cardiovascular risk factors were obtained. These included body mass index (BMI), non-fasting triglycerides, blood pressure (systolic, diastolic), total cholesterol, total cholesterol to HDL cholesterol ratio, LDL cholesterol, HDL cholesterol, non-fasting glucose, HOMA-IR, and C-reactive protein (CRP).

### Statistical analyses

All measures were normally distributed (i.e., skewness, kurtosis) except for total cholesterol/HDL ratio and CRP. Due to skewness, total cholesterol/HDL ratio was log_10_ transformed. Moderately elevated CRP (≥ 1 mg/L) has been used as a predictor of increased risk for cardiovascular disease in the general population [39]. Given that the continuous CRP variable showed a non-normal distribution, we used the clinically validated cut-off of CRP of ≥ 1 as elevated and created a dichotomized CRP variable. Pairwise deletion was used for missing data, although missing data was minimal (< 5%) in the dataset. First, an independent-samples *t*-test was conducted to investigate mean differences for the UCLA loneliness score and COVID-19-related loneliness item across substance use (*N* = 103). Then, zero-order correlations were conducted to examine associations among loneliness and markers of cardiovascular risk in the cardiovascular subsample (*n* = 56). Guided by the results of the *t*-tests and bivariate correlations, linear and logistic regressions were conducted examining the associations of loneliness, substance use, and markers of cardiovascular risk. Covariate selection was guided by prior research: older age is associated with greater loneliness severity, and loneliness can differ across the lifespan [40, 41]. Additionally, even among those receiving suppressive ART, HIV is associated with increased cardiovascular risk due to persistent inflammation causing modifications in lipid profiles [42]. Thus, linear regressions examining the associations of loneliness with cardiovascular risk adjusted for age, HIV status, and methamphetamine use. We also examined the associations of the interaction of loneliness with methamphetamine use with markers of cardiovascular risk. Levene’s test and standardized residuals were examined to ensure that equality of variances and normality of errors assumptions were met. Cook’s distance was also used to identify outliers in these models. For the logistic regression of loneliness predicting the dichotomous outcome of screening positive for a stimulant use disorder, we adjusted for age and HIV status. Goodness-of-fit was determined by using Hosmer-Lemeshow tests and examining AIC/BIC statistics. A smaller-is-better approach was taken for the goodness-of-fit statistics and a non-significant *p* value for the Hosmer-Lemeshow tests indicated good model fit. The UCLA loneliness total score was standardized for logistic regression models to ease interpretation. Statistical analyses were completed using R version 4.2.1 [43].

## Results

The total sample included 103 participants with a mean age of 38.6 (SD = 12) years old. Over half of the sample (56.3%) identified as Hispanic/Latino; 30.1% identified as non-Hispanic White; 10.7% were Black/African American; and 2.9% identified as other or multiracial. In the total sample (*N* = 103), 54 participants reported no stimulant use (52.4%), 49 participants reported stimulant use (47.60%), 63 participants (61.20%) were HIV-negative, and 40 participants (38.80%) were HIV-positive. In the cardiovascular sample (*n* = 56), 30 participants reported no stimulant use (53.60%), 26 participants reported stimulant use (46.40%), 36 participants (64.30%) were HIV-negative, and 20 participants (35.70%) were HIV-positive. Statin use was reported in 5.8% in the total sample and 3.6% of participants in the cardiovascular subsample. Statin use was relatively consistent across the groups. In the total sample (*N* = 103), 6 participants reported statin use. In the cardiovascular subsample (*n* = 56), 2 participants reported statin use in the non-methamphetamine use group and 1 participant reported statin use in the methamphetamine use group. Approximately 26% (*n* = 27) of the sample, 6 screened positive for a stimulant disorder on the ASSIST, and 14 of those participants were HIV positive. In the logistic regression analyses after adjusting for age and HIV status, each standard deviation increase in loneliness was 1.68 (95% CI = 1.03–2.87) greater odds of screening positive for a stimulant use disorder on the ASSIST. The final model, adjusting for age and HIV status, yielded the lowest AIC/BIC values and best fit for interpretation. The Hosmer–Lemeshow test yielded a non-significant *p*-value (*p* > .05).

The independent-samples *t*-test conducted to investigate mean differences for the UCLA loneliness score and COVID-19-related loneliness item across substance use revealed SMM who used methamphetamine (*t*(101) = 2.03, *p* < .05; *d* = .42) and those that screened positive for a moderate-to-severe stimulant use disorder (*t*(101) = 2.07, *p* < .05; *d* = .46) had significantly higher mean loneliness scores compared to their counterparts. There were no significant differences for COVID-19-associated loneliness across stimulant groups or those that screened positive for a moderate-to-severe stimulant use disorder (*p* > .05). As shown in Table 1, loneliness showed negative bivariate associations with total cholesterol and LDL cholesterol in the cardiovascular subsample (*n* = 56). Loneliness did not significantly correlate with BMI, non-fasting triglycerides, blood pressure, tobacco use, non-fasting glucose, or HOMA-IR. No differences were found for loneliness or loneliness associated with COVID-19 for those with elevated CRP compared to non-elevated CRP levels (*p* > .05).

In linear regression analyses, the associations of loneliness with lower LDL and total cholesterol were moderated by methamphetamine use (see Tables 2 and 3). Among SMM who used methamphetamine, higher loneliness was significantly associated with lower LDL cholesterol (*β* = − .55, *p* < .01) and lower total cholesterol (*β* = − .51, *p* < .01). In contrast, the associations of loneliness with LDL cholesterol (*β* = − .004, *p* = .99) and total cholesterol (*β* = .02, *p* = .94) were not significant among SMM who did not use methamphetamine. The negative associations of loneliness with LDL and total cholesterol were observed only among SMM who used methamphetamine.

## Discussion

This cross-sectional study highlights the importance of examining the intersection of loneliness and stimulant use in the era of COVID-19. Greater loneliness was associated with screening positive for a stimulant use disorder and lower LDL and total cholesterol in bivariate analyses. We additionally observed that the negative association of loneliness with lower cholesterol parameters was apparent only among SMM who used methamphetamine. These findings are in line with previous literature that have reported lower levels of total cholesterol and triglycerides found among methamphetamine users [44].

The decrease in LDL noted in this study could be explained by abnormal metabolic activity and nutritional status linked to the well-established effects of methamphetamine use on appetite and body mass index (BMI) in previous studies [44–46]. Considering methamphetamine’s observed associations with cholesterol, there are further important medical consequences of methamphetamine use, including cardiovascular risk, higher risk of HIV acquisition, increased risk of HIV progression, and mortality due to stimulant overdose [47]. In fact, national data trend has shown that overdose mortalities involving stimulants have increased by 43% from 2015 to 2019, and that overdose mortalities are more likely with riskier methods of use (i.e., via methamphetamine injection) which is found to be highest among SMM [47]. In another retrospective study using the US National Inpatient Sample database, which encompassed more than 35 million participants and approximately 180,000 individuals who reported methamphetamine use, it was found that methamphetamine users had a 27% increased risk of sudden cardiac death [48]. Finally, although cholesterol is considered as one piece of risk for cardiovascular disease, it could additionally be a key factor in elucidating behavioral (i.e., dietary habits, appetite suppression, substance use) and possible lipid changes linked to chronic social stressors such as loneliness.

In addition, animal studies have documented that social isolation and chronic social stress accelerate atherogenesis, and that atherogenesis in stressed animals is independent of serum lipid levels [49–51]. A previous animal study exposed monkeys to high levels of social stress and fed them a low-fat, low-cholesterol diet [50]. Those in the high social stressed groups developed atherosclerosis compared to non-socially stressed monkeys despite the absence of elevated serum lipids. Loneliness-associated atherosclerosis in socially isolated animals and humans may involve underlying mechanisms, including behavioral alterations such as physical inactivity, overactivation of the sympathetic nervous system (SNS) and hypothalamic-pituitary-adrenal (HPA) axis, vascular inflammation, and oxidative stress [51–53]. Therefore, traditional cardiovascular risk factors in this population would not fully account for the increased CVD risk observed in the setting of higher syndemic burden [54]. In fact, prior research indicates that methamphetamine use may reduce traditional cardiovascular risk factors (e.g., obesity, cholesterol) due to its effects on appetite suppression [54]. However, methamphetamines are known to have cardiotoxic effects [55]. For example, methamphetamine use has direct and indirect effects on cardiovascular health through inflammatory and immunologic pathways and is associated with cellular and structural changes of the heart—increasing risk of for cardiac arrhythmias and cardiac myopathy [54]. Subsequently, key stressors such as loneliness and substance use behaviors such as methamphetamine use amplifies cardiovascular risk [54]. Therefore, it is essential that behavioral factors are both assessed and treated to mitigate cardiovascular risk.

This cross-sectional study further underscores the need for clinical research to develop and test interventions targeting loneliness, particularly among SMM with stimulant use disorders. Programs targeted at stimulant using SMM are currently limited to the use of interventions that include methods such as cognitive behavioral therapy (CBT) or contingency management (CM) techniques [57, 58], integrating these available evidence-based methods with group-based behavioral activation models [59] or Zoom-based evidence-based group programs [60]. These methods may successfully provide more comprehensive programs for stimulant using SMM that simultaneously report loneliness and isolation. Although there is empirical evidence of the usefulness of interventions to reduce loneliness among SMM, more clinical research is needed specifically for SMM living with HIV and stimulant users.

### Limitations

First, the current study leveraged cross-sectional data from a 6-month prospective cohort study. Due to the cross-sectional design, the study cannot infer directionality and causality between loneliness, methamphetamine use, and cardiovascular risk in this sample of SMM. Second, apart from the COVID-19-specific loneliness question, the UCLA Loneliness Scale does not include a time anchor, meaning that we cannot determine whether participants were always lonely or became lonely during a particular phase in their life (e.g., after ending a relationship, during a mid-life crisis, moving to a new city). Contextualizing loneliness with time anchors is an important next step for longitudinal research to disentangle potential causality for the associations presented here. Third, data were not collected on additional health-related behaviors including physical activity, diet, and sleep. These variables would provide insight on predictors of cardiometabolic risk in this sample, as health-related behaviors may partially explain the relationship between loneliness and cholesterol. Future research should consider these potential confounding or mediation effects. Fourth, several of the included cardiovascular risk measures reflect circulating lipid levels that were non-fasting, which has the potential to influence the findings presented here. Research is needed to employ non-fasting measures to support the results discussed in this study. Fifth, this study was not able to quantify methamphetamine dosage of the participants. Methamphetamine use’s cardiotoxic effects have been found to persist in animal models across administration doses (acute, chronic, and binge administration) [56]. It is possible that the effects presented here would be more pronounced with heavier use compared to participants with less frequent use. A future study could quantify methamphetamine use, administration (e.g., chronic, acute, binge), or stimulant type to compare the indirect effect of dosage response of loneliness to cardiovascular risk. These data would add richness and context to future studies that examine these relationships. Lastly, the sample size presented here is modest and cross-sectional data was used. Longitudinal studies with sufficient statistical power are needed to unravel the complex, potentially bidirectional associations among loneliness, stimulant use, and cardiovascular risk.

### Implications for Research and Practice

These preliminary data can guide efforts to unravel the complex relationships between loneliness, stimulant use, and cholesterol levels impacting cardiovascular risk among SMM. These findings warrant interventional research to examine how reductions in loneliness among SMM may reduce stimulant use, which has important implications for increased HIV prevention and decreased cardiovascular risk. Additionally, there is a necessity for increased awareness on the implications of loneliness and methamphetamines on cardiovascular health as traditional risk factor estimators may be misleading in underestimating their actual risk. Future studies should include individuals who use methamphetamine to increase the generalizability and understanding of loneliness and cardiovascular risk, and to expand upon the findings presented here.

## Figures and Tables

**Table 1:** Bivariate correlations among loneliness and markers in cardiovascular risk subset sample, *n* = 56

Variable	1	2	3	4	5	6	7	8	9	10	11	12	13
1. UCLA Loneliness Scale	1												
2. COVID-19 loneliness	0.40**	1											
3. Triglycerides	−0.10	−0.14	1										
4. Total cholesterol	−0.27*	−0.25	0.26	1									
5. HDL cholesterol	0.13	0.14	−0.51**	−0.01	1								
6. LDL cholesterol	−0.28*	−0.25	0.01	0.91***	−0.17	1							
7.Total/HDL ratio	−0.26	−0.25	0.57***	0.57***	−0.81***	0.64***	1						
8. BMI	0.12	−0.11	0.11	−0.04	−0.27*	0.01	0.21	1					
9. Systolic BP	0.06	0.01	0.14	−0.03	−0.19	−0.02	0.15	0.39*	1				
10. Diastolic BP	0.05	0.02	0.16	0.13	−0.24	0.16	0.32	0.27*	0.81***	1			
11. Tobacco use	0.11	0.05	−0.02	−0.00	−0.10	0.05	0.07	−0.04	0.17	0.26	1		
12. Glucose	−0.04	0.07	0.06	0.07	−0.17	0.11	0.17	0.12	0.15	0.07	−0.04	1	
13. HOMA-IR	0.20	0.08	0.36**	0.01	−0.29*	−0.04	0.26	0.38**	0.41**	0.41**	0.28	0.11	1

Analyses were zero-order correlations

* *p* < .05;

** *p* < .01;

*** *p* < .001

Total cholesterol/HDL ratio was log_10_ transformed

**Table 2:** Linear regression models testing the association of loneliness and LDL cholesterol controlling for HIV status, age, and methamphetamine use, *N* = 56

	Model 1 *B* (95% CIs)	Model 2 *B* (95% CIs)	Model 3 *B* (95% CIs)
UCLA Loneliness Scale	− 1.82* [− 3.25, − 0.39]	− 1.92** [− 3.39, − 0.45]	− 2.93** [− 4.68, − 1.18]
HIV status		− 7.04 [− 26.10, 12.03]	− 8.51 [− 27.09, 10.08]
Age		0.22 [− 0.54, 0.97]	0.29 [− 0.45, 1.02]
Methamphetamine use		8.22 [− 11.21, 27.65]	− 73.44 [− 156.95, 10.07]
Loneliness * Methamphetamine use			3.07* [0.01, 6.13]
*R* ^2^	.108*	.132	.198*
Adj. *R*^2^	0.091*	0.064	.117*
*F*	6.538	1.944	2.462
*p* value	0.013	0.117	0.045

* indicates *p* < .05.

** indicates *p* < .01

**Table 3:** Linear regression models testing the association of loneliness and total cholesterol controlling for HIV status, age, and methamphetamine use, *N* = 56

	Model 1 *B* (95% CIs)	Model 2 *B* (95% CIs)	Model 3 *B* (95% CIs)
UCLA Loneliness Scale	− 1.80* [− 3.27, − 0.33]	− 1.90* [− 3.39, − 0.42]	− 3.00** [− 4.75, − 1.25]
HIV status		− 15.88 [− 35.09, 3.33]	− 17.48 [− 36.08, 1.12]
Age		0.37 [− 0.39, 1.13]	0.44 [− 0.29, 1.18]
Methamphetamine use		9.24 [9.24, 28.82]	− 79.48 [− 163.06, 4.11]
Loneliness * Methamphetamine use			3.34* [0.28, 6.40]
*R* ^2^	.100*	.164	.237*
Adj. *R*^2^	0.084*	0.099	.161*
*F*	6.023	2.505	3.11
*p* value	0.017	0.053	0.016

* indicates *p* < .05.

** indicates *p* < .01

## Data Availability

De-identified data from this project are available upon request.
